# The Pretreatment Neutrophil-to-Eosinophil Ratio Can Predict Immune-Related Adverse Events and Outcomes in Patients With Advanced Urothelial Carcinoma Treated With Immune Checkpoint Inhibitors

**DOI:** 10.7759/cureus.89650

**Published:** 2025-08-08

**Authors:** Keita Kobayashi, Shigeru Sakano, Hiroaki Matsumoto, Masahiro Tsuchida, Yasuhide Tei, Kazuhiro Nagao, Kazuo Oba, Seiji Kitahara, Seiji Yano, Koji Shiraishi

**Affiliations:** 1 Department of Urology, Graduate School of Medicine, Yamaguchi University, Ube, JPN; 2 Department of Urology, Kokura Memorial Hospital, Kitakyushu, JPN; 3 Department of Urology, Yamaguchi Prefectural Grand Medical Center, Hofu, JPN; 4 Department of Urology and Nephrology, Tokuyama Central Hospital, Shunan, JPN; 5 Department of Urology, Kanmon Medical Center, Shimonoseki, JPN; 6 Department of Urology, Shuto General Hospital, Yanai, JPN; 7 Department of Urology, Saiseikai Yamaguchi General Hospital, Yamaguchi, JPN; 8 Department of Urology, Nagato General Hospital, Nagato, JPN; 9 Department of Urology, Masuda Red Cross Hospital, Masuda, JPN

**Keywords:** advanced urothelial carcinoma, immune-checkpoint inhibitors, immune-related adverse event (irae), neutrophil-to-eosinophil ratio, overall survival (os), progression - free survival

## Abstract

Introduction

Currently, treatment regimens incorporating immune checkpoint inhibitors (ICIs) are the standard of care for locally advanced or metastatic urothelial carcinoma (la/mUC). This study aimed to investigate the association between the neutrophil-to-eosinophil ratio (NER) and the occurrence of immune-related adverse events (irAEs) as well as treatment outcomes.

Methods

This multicenter retrospective study examined patients with la/mUC treated with ICIs between January 2017 and December 2022.

Results

A total of 243 patients with la/mUC were analyzed: 207 who received pembrolizumab after chemotherapy and 36 who received avelumab as maintenance therapy. In the overall cohort, the median progression-free survival (PFS) from the initiation of ICIs was 5.3 months, while the median overall survival (OS) was 23.9 months. Grade 2 or higher irAEs were observed in 72 patients (29.6%), whereas grade 3 or higher events were identified in 31 patients (12.8%). In univariate analysis, the neutrophil-to-lymphocyte ratio (NLR), platelet-to-lymphocyte ratio (PLR), NER, and systemic immune-inflammation index (SII) at the initiation of treatment were identified as predictive factors for grade 2 or higher irAEs. In multivariate analysis, NER was found to be an independent predictive factor. Patients with NER of ≤44 at the initiation of treatment had a significantly higher incidence of grade 2 or higher irAEs than those with NER >44 (36.7% vs. 20.2%; p=0.009). However, they also exhibited significantly longer PFS (p=0.003) and OS (p<0.001).

Conclusions

In la/mUC, pretreatment NER may serve as a predictive marker for irAEs and treatment outcomes in patients receiving ICIs.

## Introduction

Platinum-based chemotherapy is the mainstay treatment for locally advanced or metastatic urothelial carcinoma (la/mUC). In 2017, the efficacy of pembrolizumab as a second-line treatment after chemotherapy was reported [1]. In 2021, avelumab was shown to be an effective maintenance therapy after chemotherapy [2]. In 2023, the addition of nivolumab to the conventional first-line chemotherapy regimen of gemcitabine and cisplatin was shown to provide a survival benefit [3]. Subsequently, in 2024, the combination therapy of enfortumab vedotin, an antibody-drug conjugate targeting Nectin-4, and pembrolizumab was shown to be superior in overall survival (OS) compared to gemcitabine and cisplatin [4]. The current standard treatment for la/mUC primarily consists of immune checkpoint inhibitors (ICIs), either as monotherapy or in combination with other therapies. The effective management of ICIs plays a crucial role in optimizing treatment outcomes.

Similar to other cancer types, studies have reported that patients with urothelial carcinoma who develop immune-related adverse events (irAEs) demonstrate improved objective response rates, progression-free survival (PFS), and OS [5,6]. Although one study reported an association between irAE occurrence and treatment efficacy regardless of treatment duration [7], retrospective studies have suggested that prolonged treatment duration due to therapeutic response may increase the likelihood of irAE development with ICIs. Various blood markers, including absolute lymphocyte count, eosinophil count, neutrophil-to-lymphocyte ratio (NLR), and platelet-to-lymphocyte ratio (PLR), have been reported as predictors of irAEs [8]. As in other cancer types, the neutrophil-to-eosinophil ratio (NER) has been investigated as a prognostic marker for ICIs therapy in urothelial carcinoma, albeit with limited cancer types [9,10]. Although a study with a small sample size suggested NER as a predictive marker for irAEs in renal cell carcinoma [11], no such reports are available for urothelial carcinoma.

In this study, we investigated the association between pretreatment NER and the incidence of irAEs as well as survival outcomes in patients with la/mUC who received ICIs following platinum-based chemotherapy, based on the hypothesis that NER may have a dual role as both a prognostic factor and a predictive marker for irAE development in urothelial carcinoma.

## Materials and methods

Study population

This multicenter, retrospective study was conducted across 15 institutions. This study included patients with la/mUC who received ICIs between January 2017 and December 2022. All patients underwent platinum-based chemotherapy before receiving ICIs. The observation period was extended from the initiation of ICI treatment to March 2023. This study was conducted per the ethical guidelines for life sciences and medical research involving human subjects. The study protocol was disclosed to potential participants, and the opportunity to opt out was provided, thereby indicating the need for individual informed consent. This study was approved by the Research Ethics Committee of Yamaguchi University Hospital (ID: 2023-062).

Data collection and determination of cutoff values

Patient and tumor characteristics, details of ICIs treatment, and various blood test data were collected from the medical records.　Adverse events were assessed by the treating physicians using the Common Terminology Criteria for Adverse Events, version 5.0. [12]. NLR was calculated by dividing the neutrophil count by the lymphocyte count, PLR by dividing the platelet count by the lymphocyte count, and NER by dividing the neutrophil count by the eosinophil count. The systemic immune-inflammation index (SII) was calculated by multiplying the platelet count by the neutrophil count and dividing the result by the lymphocyte count. The modified Glasgow Prognostic Score (mGPS) was defined as follows: a score of 0 was assigned to patients with C-reactive protein (CRP) ≤0.5 mg/dL and serum albumin ≥3.5 g/dL; a score of 1 was assigned to those with either CRP >0.5 mg/dL or serum albumin <3.5 g/dL; and a score of 2 was assigned to those with both CRP >0.5 mg/dL and serum albumin <3.5 g/dL. The prognostic nutritional index was calculated by adding the value obtained by multiplying the serum albumin level by 10 to the value obtained by multiplying the lymphocyte count by 0.005. The cutoff values for various blood parameters, inflammatory markers, and nutritional indices were determined individually using receiver operating characteristic (ROC) curve analysis based on the presence or absence of grade 2 or higher irAEs.

Statistical analysis

The differences between the two groups were evaluated for statistical significance using the Wilcoxon signed-rank test and Pearson's chi-square test. PFS was defined as the time from the initiation of ICIs therapy to disease progression or death from any cause. OS was defined as the time from the initiation of ICI therapy to death from any cause. Kaplan-Meier curves were constructed, and statistical significance for PFS and OS was assessed using the log-rank test. To compare survival outcomes according to the presence or absence of grade ≥2 AEs, a landmark analysis was performed, including only those patients who had neither experienced disease progression nor died by week 12, to exclude those who discontinued treatment due to early disease progression. Univariate and multivariate logistic regression analyses were performed to evaluate the predictive factors. All statistical analyses were conducted using JMP Pro version 18.1.2 (SAS, Cary, NC), with a two-sided significance level of 5% and a confidence interval (CI) set at 0.95.

## Results

A total of 243 patients were included in this study: 207 received pembrolizumab and 36 received avelumab. Detailed patient characteristics, treatment information, and mGPS are presented in Table 1, while baseline blood test results and inflammatory marker levels before ICI treatment for la/mUC are shown in Table 2. The median duration of ICI administration was 4.8 months (IQR: 2.0-13.0), and the median observation period was 14.4 months (IQR: 5.9-24.3). The median PFS and OS for all patients treated with ICIs were 5.3 months and 23.9 months, respectively. Among patients treated with pembrolizumab, the median PFS was 4.5 months, and the median OS was 20.4 months. In contrast, for those who received avelumab, the median PFS was 12.2 months, and the median OS was 29.1 months.

**Table 1:** Patient and tumor characteristics and mGPS in patients with la/mUC treated with ICIs (n=243) mGPS: modified Glasgow Prognostic Score; la/mUC: locally advanced or metastatic urothelial carcinoma; ICI: immune checkpoint inhibitor; IQR: interquartile range; ECOG PS: Eastern Cooperative Oncology Group Performance Status; UTUC: upper urinary tract urothelial carcinoma; UC: urothelial carcinoma; GC: gemcitabine and cisplatin; Gcarbo: gemcitabine and carboplatin; MVAC: methotrexate and vinblastine, doxorubicin, cisplatin; BOR: best overall response; CR: complete response; PR: partial response; SD: stable disease

Variables	Category	Values
Age, years, median (IQR)		74 (69-80)
Sex, n (%)	Male	179 (74%)
	Female	64 (26%)
ECOG PS, n (%)	0	204 (84%)
	1	31 (13%)
	2	6 (2%)
	≥3	2 (1%)
History of smoking, n (%)	No	90 (37%)
	Yes	141 (58%)
	Unknown	12 (5%)
Primary site, n (%)	Bladder	106 (44%)
	UTUC	106 (44%)
	Both	30 (11%)
	Unknown	1 (1%)
Primary removal, n (%)	No	121 (50%)
	Yes	122 (50%)
Radiotherapy to primary site, n (%)	No	197 (81%)
	Yes	46 (19%)
Histological type, n (%)	UC	170 (70%)
	UC variant	24 (10%)
	Other	16 (7%)
	Unknown	33 (13%)
Visceral metastasis, n (%)	No	134 (55%)
	Yes	109 (45%)
Liver metastasis, n (%)	No	202 (83%)
	Yes	41 (17%)
Prior chemotherapy regimen, n (%)	GC	157 (65%)
	GCarbo	64 (26%)
	MVAC	3 (1%)
	Other	19 (8%)
Number of prior chemotherapy cycles, median (range)		3 (1-13)
BOR to prior chemotherapy, n (%)	CR	8 (3%)
	PR	69 (29%)
	SD	78 (32%)
	PD	58 (24%)
	Unknown	30 (12%)
Content of ICI, n (%)	Avelumab	36 (15%)
	Pembrolizumab	207 (85%)
mGPS, n (%)	0	100 (43%)
	1	68 (30%)
	2	62 (27%)

**Table 2:** Baseline blood test results and inflammatory marker levels at ICI initiation in la/mUC patients ICI: immune checkpoint inhibitor; la/mUC: locally advanced or metastatic urothelial carcinoma; IQR: interquartile range; CRP: C-reactive protein; NLR: neutrophil-to-lymphocyte ratio; PLR: platelet-to-lymphocyte ratio; NER: neutrophil-to-eosinophil ratio; SII: systemic immune-inflammation index; PNI: prognostic nutritional index

Variables	Values, median (IQR)	Reference range
Neutrophil count, /μL	3658 (626-39528)	1270-6923
Lymphocyte count, /μL)	1224 (86-5024)	545-4257
Eosinophil count, μL	107 (54-219)	0-731
Hemoglobin, g/dL	10.6 (5.7-15.5)	Male: 13.7-16.8/female: 11.6-14.8
Platelet count, ×10^4^/μL	21.1 (1.2-78.2)	15.8-34.8
Serum albumin, g/dL	3.7 (3.3-4.1)	4.1-5.1
CRP, mg/dL	0.52 (0-16.61)	0-0.14
NLR	2.96 (0.32-197)	ー
PLR	172 (12-5267)	ー
NER	33 (18-61)	ー
SII	580 (340-1193)	ー
PNI	44 (39-49)	ー

**Figure 1:**
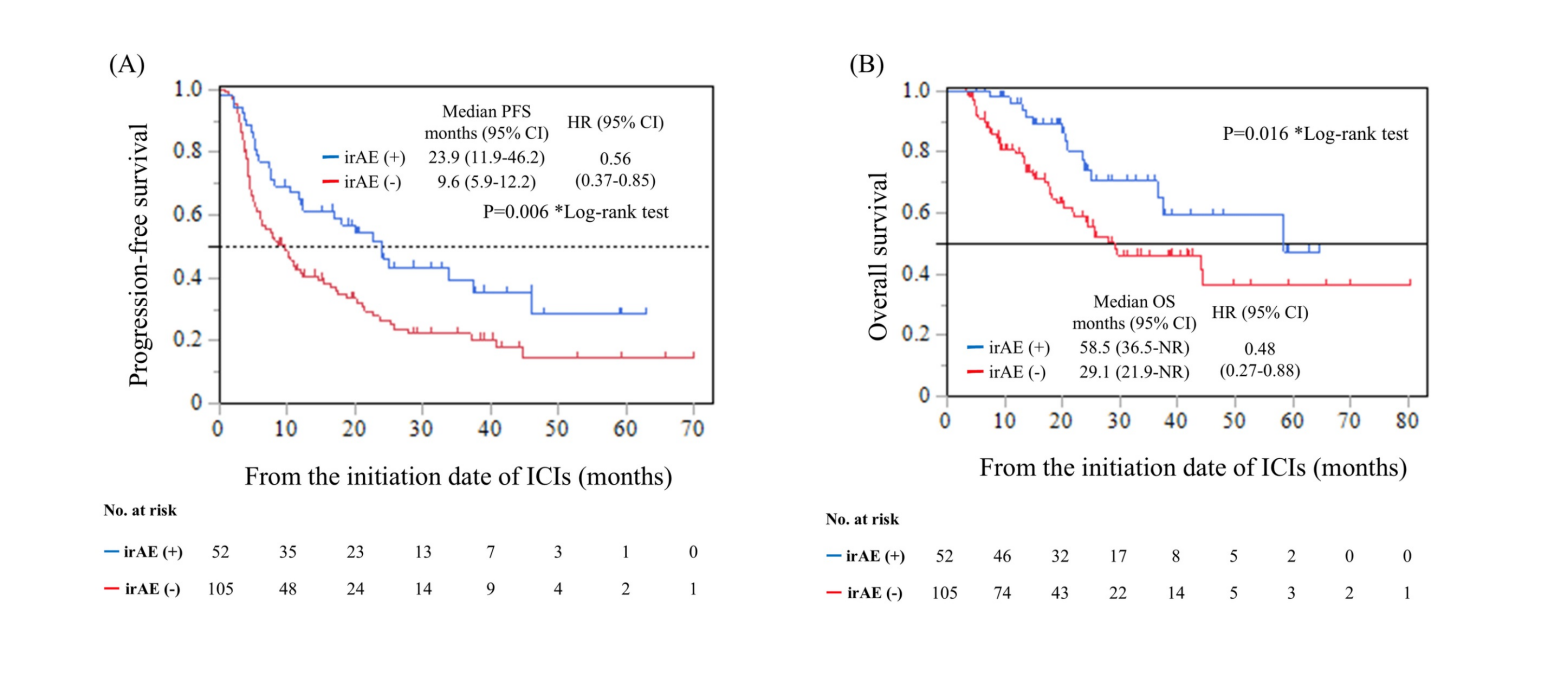
Landmark analysis of survival outcomes based on the presence or absence of Grade ≥2 irAEs in patients with la/mUC treated with ICIs: (A) PFS and (B) OS Landmark analysis was performed in 157 patients, excluding those who experienced disease progression or death within 12 weeks of ICI initiation irAE: immune-related adverse event; la/mUC: locally advanced or metastatic urothelial carcinoma; ICI: immune checkpoint inhibitor; PFS: progression-free survival; OS: overall survival; HR: hazard ratio; CI: confidence interval

A total of 72 cases of grade ≥2 irAEs (29.6%) were observed, including 60 in the pembrolizumab group (29.0%) and 12 in the avelumab group (33.3%) (Table 3). Grade ≥3 irAEs occurred in 31 patients (12.8%) (Table 3). Grade ≥2 hypothyroidism was observed in 15 patients (6.2%), interstitial lung disease in 11 patients (4.5%), and adrenal insufficiency and skin rash in eight patients (3.3%) (Table 3). In a landmark analysis of 157 patients without disease progression or death at 12 weeks, those who developed grade ≥2 irAEs had significantly longer PFS (HR: 0.56, p=0.006) and OS (HR: 0.48, p=0.016) (Figure 1).

**Table 3:** Details of treatment-related adverse events

Variables	All cases (n=243)			Avelumab (n=36)			Pembrolizumab (n=207)		
	Grade 1	Grade 2	Grade ≥3	Grade 1	Grade 2	Grade ≥3	Grade 1	Grade 2	Grade ≥3
Interstitial pneumonia, n (%)	9 (3.7%)	4 (1.6%)	7 (2.9%)	2 (5.6%)	0	0	7 (3.4%)	4 (1.9%)	7 (3.4%)
Hypothyroidism, n (%)	0	15 6.2%)	0	0	2 (5.6%)	0	0	13 (6.3%)	0
Hyperthyroidism, n (%)	0	1 (0.4%)	0	0	1 (2.8%)	0	0	0	0
Adrenal insufficiency, n (%)	0	4 (1.6%)	4 (1.6%)	0	2 (5.6%)	1 (2.8%)	0	2 (1.0%)	3 (1.4%)
Hypopituitarism, n (%)	0	1 (0.4%)	2 (0.8%)	0	1 (2.8%)	0	0	0	2 (1.0%)
Hyperglycemia, n (%)	0	0	3 (1.2%)	0	0	2 (5.6%)	0	0	1 (0.5%)
Liver dysfunction, n (%)	2 (0.8%)	1 (0.4%)	4 (1.6%)	1 (2.8%)	0	1 (2.8%)	1 (0.5%)	1 (0.5%)	3 (1.4%)
Renal dysfunction, n (%)	0	1 (0.4%)	5 (2.1%)	0	0	2 (5.6%)	0	1 (0.5%)	3 (1.4%)
Colitis, n (%)	2 (0.8%)	2 (0.8%)	4 (1.6%)	0	0	0	2 (1.0%)	2 (1.0%)	4 (1.9%)
Other gastrointestinal-related events, n (%)	1 (0.4%)	1 (0.4%)	0	0	0	0	1 (0.5%)	1 (0.5%)	0
Arthritis, n (%)	0	2 (0.8%)	1 (0.4%)	0	0	0	0	2 (1.0%)	1 (0.5%)
Skin rash, n (%)	7 (2.9%)	7 (2.9%)	1 (0.4%)	1 (2.8%)	2 (5.6%)	0	6 (2.9%)	5 (2.4%)	1 (0.5%)
Pruritus, n (%)	2 (0.8%)	1 (0.4%)	0	0	0	0	2 (1.0%)	1 (0.5%)	0
Other dermatological events, n (%)	0	2 (0.8%)	0	0	0	0	0	2 (1.0%)	0
Generalized muscle weakness, n (%)	1 (0.4%)		0	0	0	0	1 (0.5%)	2 (1.0%)	0
Infusion reaction, n (%)	0	7 (2.9%)	0	0	5 (13.9%)	0	0	2 (1.0%)	0
Others, n (%)	10 (4.1%)	4 (1.6%)	2 (0.8%)	0	0	0	10 (4.8%)	4 (1.9%)	2 (1.0%)

**Figure 2:**
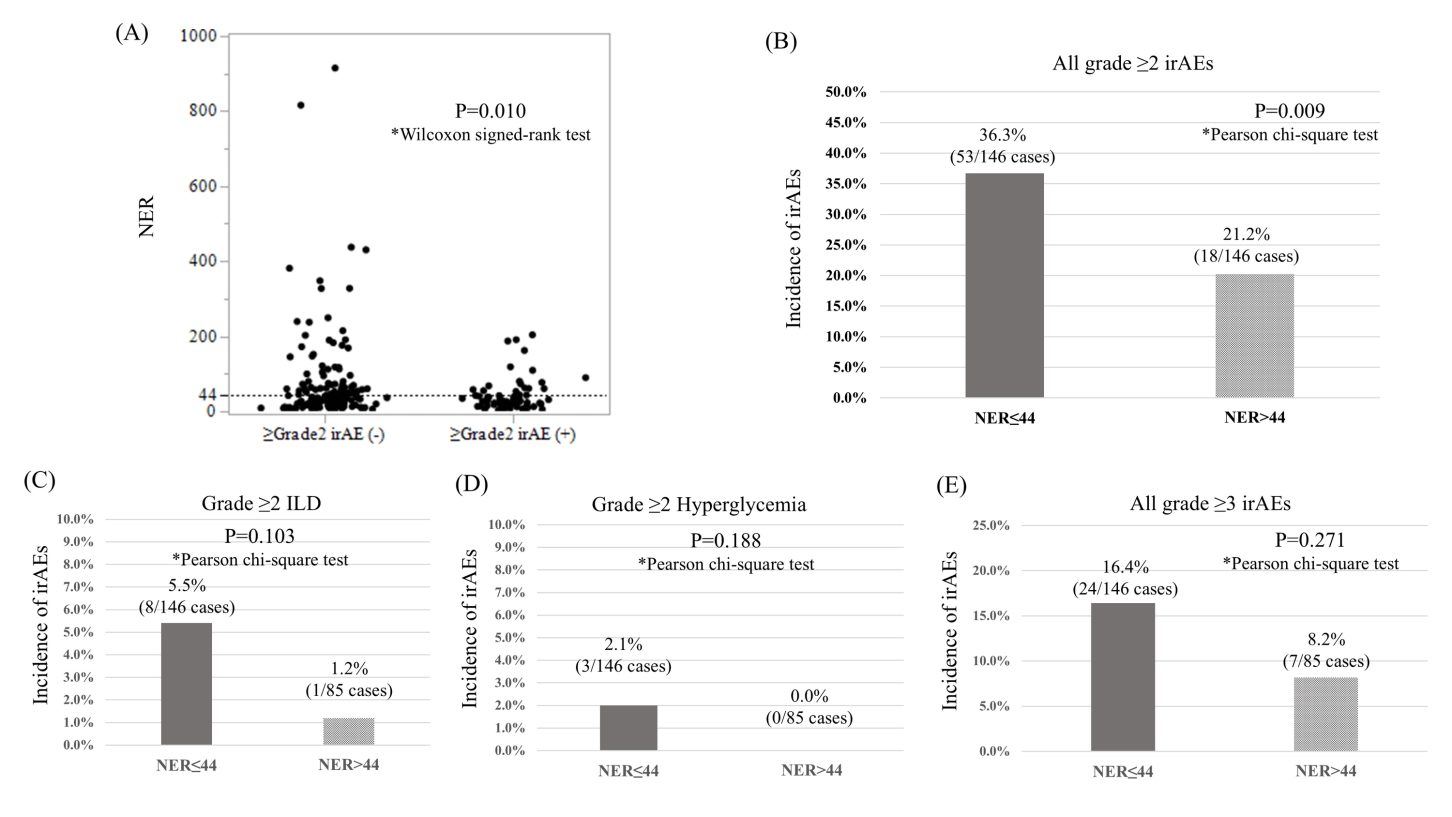
Association between the NER at the initiation of ICI therapy and the development of irAEs (A, B) Relationship between Grade ≥2 irAEs and NER; (C) Grade ≥2 ILD; (D) Grade ≥2 Hyperglycemia; (E) All Grade ≥3 irAEs. NER: neutrophil-to-eosinophil ratio; ICI: immune checkpoint inhibitor; irAE: immune-related adverse event; ILD: interstitial lung disease

Regarding patient characteristics and blood data, including inflammatory markers and nutritional indices, univariate analysis identified pretreatment NLR, PLR, NER, and SII as potential predictors of grade ≥2 irAEs (Table 4). In multivariate analysis, NER was identified as the only significant predictive factor (Table 4). The median NER in patients who developed grade ≥2 irAEs was 26.6 (IQR: 13.5-44.0), whereas in those without grade ≥2 irAEs, the median NER was significantly higher at 36.1 (IQR: 18.5-67.8) (Figure 2A). Among patients with a pre-treatment NER ≤44, 36.7% developed grade ≥2 irAEs, whereas only 20.2% of those with NER >44 experienced such events (p=0.009) (Figure 2B). Although not statistically significant, grade ≥2 interstitial lung disease (5.4% vs. 1.2%) and hyperglycemia (2.1% vs. 0%), as well as all grade ≥3 irAEs (36.3% vs. 21.2%), tended to be more frequent in patients with a NER ≤44 (Figures 2C-2E). The median NER in the pembrolizumab group was 19.9 (IQR: 9.9-53.0), while that in the avelumab group was 33.4 (IQR: 19.1-62.3), with no statistically significant difference observed between the treatment groups (p=0.052). The median change rate of the NER one month after the initiation of treatment, relative to baseline, was 0.66 (IQR: 0.39-1.14) in cases with grade ≥2 irAEs and 0.85 (IQR 0.43-1.24) in cases without such events. No significant difference was observed in the incidence of grade ≥2 irAEs between cases in which the NER decreased and those in which it increased one month after treatment initiation, compared to baseline (OR: 0.83, p=0.563).

**Table 4:** Univariable and multivariable analyses to identify factors associated with the occurrence of grade ≥2 irAEs irAE: immune-related adverse event; OR: odds ratio; CI: confidence interval; ECOG PS: Eastern Cooperative Oncology Group Performance Status; UTUC: upper urinary tract urothelial carcinoma; UC: urothelial carcinoma; ICI: immune checkpoint inhibitor; LDH: lactase dehydrogenase; CRP: C-reactive protein; NLR: neutrophil-to-lymphocyte ratio; PLR: platelet-to-lymphocyte ratio; NER: neutrophil-to-eosinophil ratio; SII: systemic immune-inflammation index; mGPS: modified Glasgow Prognostic Score; PNI: prognostic nutritional index

Variable	Category	Univariate analysis			Multivariate analysis		
		OR	95% CI	P-value	OR	95% CI	P-value
Age, years	≥75 vs. <75	1.05	0.60-1.83	0.86	ー	ー	ー
Sex	Male vs. female	0.9	0.48-1.70	0.742	ー	ー	ー
ECOG PS	≥1 vs. 0	0.67	0.29-1.45	0.318	ー	ー	ー
History of smoking	Yes vs. no	0.99	0.56-1.78	0.99	ー	ー	ー
Primary site	Bladder	Reference					
	UTUC	1.19	0.67-2.14	0.553	ー	ー	ー
	Both	0.6	0.21-1.54	0.304	ー	ー	ー
Radiotherapy to the primary site	Yes vs. no	1.34	0.67-2.63	0.401	ー	ー	ー
Visceral metastasis	Yes vs. no	0.77	0.44-1.34	0.351	ー	ー	ー
Number of prior chemotherapy cycles	≤4 vs. >4	1.79	0.90-3.76	0.097	ー	ー	ー
Content of ICI	Pembrolizumab vs. avelumab	0.82	0.39-1.79	0.601	ー	ー	ー
Hemoglobin, g/dL	<11 vs. ≥11	0.79	0.45-1.38	0.406	ー	ー	ー
CRP, mg/dL	>6.0 vs. ≤6.0	0.52	0.17-1.34	0.187	ー	ー	ー
Serum albumin, g/dL	<3.0 vs. ≥3.0	0.59	0.23-1.36	0.222	ー	ー	ー
NLR	>2.6 vs. ≤2.6	0.55	0.31-0.95	0.034	0.82	0.39-1.70	0.584
PLR	>86 vs. ≤86	0.46	0.22-0.98	0.043	0.57	0.24-1.34	0.195
NER	>44 vs. ≤44	0.47	0.25-0.86	0.015	0.52	0.27-0.98	0.042
SII	>500 vs. ≤500	0.53	0.30-0.93	0.027	0.82	0.38-1.77	0.603
mGPS	0	Reference					
	1	1.07	0.54-2.11	0.843	ー	ー	ー
	2	1.32	0.66-2.61	0.431	ー	ー	ー
PNI	>40 vs. ≤40	1.38	0.75-2.63	0.304	ー	ー	ー

**Figure 3:**
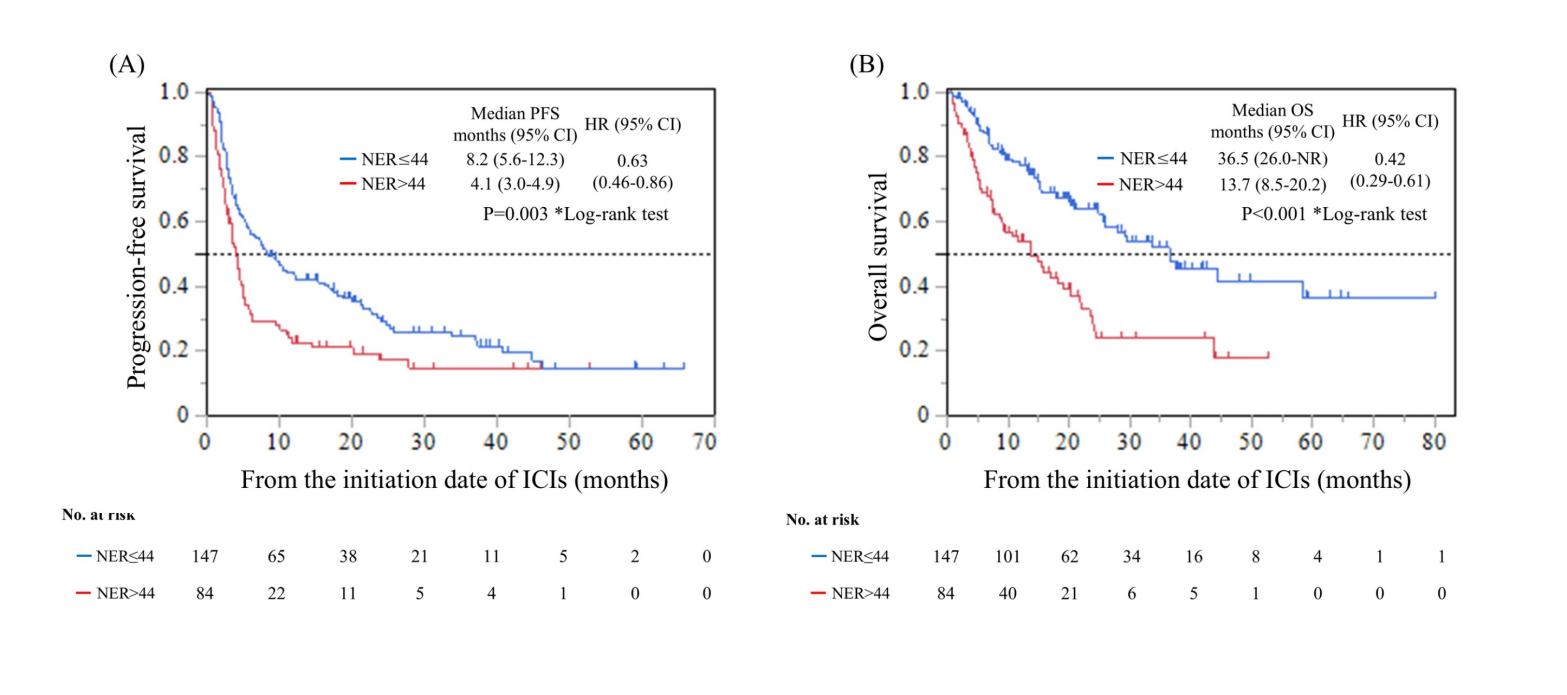
Kaplan–Meier curves based on the NER at the initiation of ICI therapy: (A) PFS; (B) OS NER: neutrophil-to-eosinophil ratio; ICI: immune checkpoint inhibitor; PFS: progression-free survival; OS: overall survival; HR: hazard ratio; CI: confidence interval

The median PFS for patients with an NER ≤44 at the start of treatment was 8.2 months (95% CI: 5.6-12.3), which was significantly longer compared to a median PFS of 4.1 months (95% CI: 3.0-4.9) for patients with an NER >44 (HR: 0.63, p=0.003) (Figure 3A). Similarly, OS was significantly longer in patients with an NER ≤44, with a median OS of 36.5 months (95% CI: 26.0-not reached), compared to a median OS of 13.7 months (95% CI: 8.5-20.2) in patients with an NER >44 (HR: 0.42, p<0.001) (Figure 3B).

## Discussion

In this multicenter retrospective study, we demonstrated that a low baseline NER ≤44 was significantly associated with both a higher incidence of grade ≥2 irAEs and longer PFS and OS in patients with la/mUC treated with ICIs. These findings suggest that NER may serve as a valuable predictive biomarker for both irAE risk and the therapeutic efficacy of ICI treatment.

An association between irAE development and favorable oncological outcomes has been reported in several malignancies, including urothelial carcinoma [13,14]. On the other hand, some reports have indicated a weak correlation between irAEs and the efficacy of ICIs [15,16]. The development of irAEs can occur even in the later phases of ICI therapy, and the duration of treatment may influence the incidence of irAEs. In retrospective studies, patients who experienced early disease progression or death often did not have sufficient time to develop irAEs, resulting in a poorer prognosis. Consequently, patients without irAEs may be disproportionately classified as having poor outcomes. In the present study, we addressed this potential bias by conducting a landmark analysis limited to patients who did not experience disease progression or death within the first 12 weeks after ICI initiation. In the selected cohort, we observed that patients who developed grade 2 or higher irAEs had significantly longer PFS and OS, indicating a potential association between irAE occurrence and favorable oncologic outcomes.

An association between NER and oncological outcomes has been reported in various malignancies, including urothelial carcinoma [9,17,18]. However, studies investigating the relationship between NER and irAE development remain limited [11,19]. To date, no such reports exist in the context of ICI therapy for urothelial carcinoma. In this study, we conducted multivariate analyses of various inflammation-related markers, including NLR, PLR, and SII. Of these, only the NER emerged as an independent predictor of grade ≥2 irAEs. Furthermore, NER was also significantly associated with survival outcomes. These findings suggest the potential utility of NER as a comprehensive biomarker for patients with la/mUC receiving ICIs.

Neutrophils have been reported to promote tumor progression through immunosuppressive and pro-inflammatory mechanisms, whereas eosinophils have been shown to mediate antitumor immunity by recruiting cytotoxic T cells and secreting cytokines such as interleukin-5 and granulocyte-macrophage colony-stimulating factor [20,21]. Therefore, a low NER may reflect an immune environment that is not only more prone to irAE development but also potentially indicative of a favorable response to ICIs. These findings suggest that NER may serve as a more comprehensive biomarker than other inflammatory indices, as it simultaneously captures protumor inflammatory activity driven by neutrophils and antitumor immune responses mediated by eosinophils-dual aspects of immune dynamics that may underlie its superior predictive power for both irAE incidence and survival outcomes.

In recent years, the systemic treatment landscape for la/mUC has undergone rapid changes. ICIs have been established as key agents and are now being incorporated into combination regimens and treatment sequences [3,4]. With the advent of combination therapies, adverse events have become more frequent and complex, making the ability to predict irAEs an increasingly important clinical challenge. Although no statistically significant differences were observed owing to the limited sample size, patients with low NER tended to exhibit higher frequencies of interstitial lung disease, hyperglycemia, and grade ≥3 irAEs. This suggests that severe irAEs associated with ICIs may be predicted in advance. The NER is a readily accessible and cost-effective biomarker that can be used in routine clinical practice. In patients with la/mUC, it has the potential to serve as a dual-purpose marker for predicting the risk of irAEs and oncological outcomes. In particular, patients with a low NER may be more likely to benefit from ICIs and require closer monitoring of irAEs. This suggests that NER can contribute to the implementation of personalized treatment strategies in clinical practice.

This study has several limitations. The retrospective design may introduce the potential for selection bias, and eosinophil counts may have been influenced by factors unrelated to cancer or ICI therapy, such as allergic conditions or corticosteroid use. Additionally, the relatively small number of patients who received avelumab (n=36) as maintenance therapy limits the ability to draw definitive conclusions regarding the applicability of NER across different ICI regimens. Hence, validation in larger-scale prospective studies is required to confirm the predictive accuracy and generalizability of NER in this population.

## Conclusions

NER may serve as a biomarker for predicting the development of irAEs and oncological outcomes in patients with la/mUC. Even in the context of combination therapies, where adverse events are often complex, the use of NER as a biomarker may enable more effective monitoring and management of irAEs. However, large-scale prospective studies and validation cohorts are needed to confirm these findings and establish the clinical utility of NER.
